# Implementation of diagnostic stewardship in two surgical ICUs: Time for a blood-culture change

**DOI:** 10.1017/ash.2023.221

**Published:** 2023-09-29

**Authors:** Jessica Seidelman, Rebekah Moehring, Erin Gettler, Jay Krishnan, Christopher Polage, Margaret Murphy, Rachel Jordan, Sarah Lewis, Becky Smith, Deverick Anderson, Nitin Mehdiratta

## Abstract

**Background:** Blood cultures are commonly ordered for patients with low risk of bacteremia. Liberal blood-culture ordering increases the risk of false-positive results, which can lead to increased length of stay, excess antibiotics, and unnecessary diagnostic procedures. We implemented a blood-culture indication algorithm with data feedback and assessed the impact on ordering volume and percent positivity. **Methods:** We performed a prospective cohort study from February 2022 to November 2022 using historical controls from February 2020 to January 2022. We introduced the blood-culture algorithm (Fig. 1) in 2 adult surgical intensive care units (ICUs). Clinicians reviewed charts of eligible patients with blood cultures weekly to determine whether the blood-culture algorithm was followed. They provided feedback to the unit medical directors weekly. We defined a blood-culture event as ≥1 blood culture within 24 hours. We excluded patients aged <18 years, absolute neutrophil count <500, and heart and lung transplant recipients at the time of blood-culture review. **Results:** In total, 7,315 blood-culture events in the preintervention group and 2,506 blood-culture events in the postintervention group met eligibility criteria. The average monthly blood-culture rate decreased from 190 blood cultures per 1,000 patient days to 142 blood cultures per 1,000 patient days (*P* < .01) after the algorithm was implemented. (Fig. 2) The average monthly blood-culture positivity increased from 11.7% to 14.2% (*P* = .13). Average monthly days of antibiotic therapy (DOT) was lower in the postintervention period than in the preintervention period (2,200 vs 1,940; *P* < .01). (Fig. 3) The ICU length of stay did not change before the intervention compared to after the intervention: 10 days (IQR, 5–18) versus 10 days (IQR, 5–17; *P* = .63). The in-hospital mortality rate was lower during the postintervention period, but the difference was not statistically significant: 9.24% versus 8.34% (*P* = .17). The all-cause 30-day mortality was significantly lower during the intervention period: 11.9% versus 9.7% (*P* < .01). The unplanned 30-day readmission percentage was significantly lower during the intervention period (10.6% vs 7.6%; *P* < .01). Over the 9-month intervention, we reviewed 916 blood-culture events in 452 unique patients. Overall, 74.6% of blood cultures followed the algorithm. The most common reasons overall for ordering blood cultures were severe sepsis or septic shock (37%), isolated fever and/or leukocytosis (19%), and documenting clearance of bacteremia (15%) (Table 1). The most common indications for inappropriate blood cultures were isolated fever and/or leukocytosis (53%). **Conclusions:** We introduced a blood-culture algorithm with data feedback in 2 surgical ICUs and observed decreases in blood-culture volume without a negative impact on ICU LOS or mortality rate.

**Figure:**
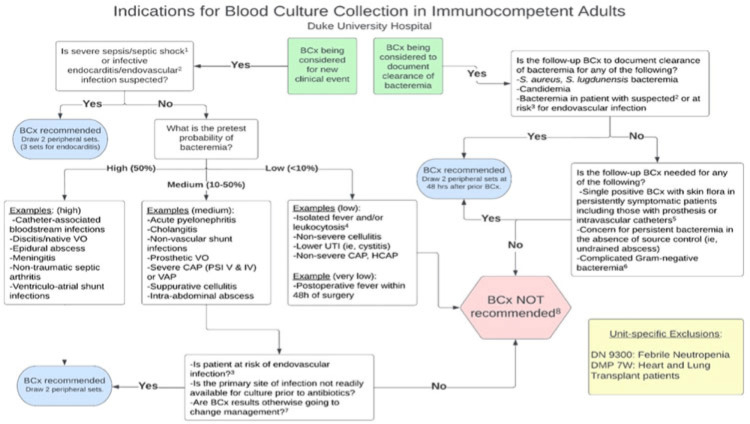

**Figure:**
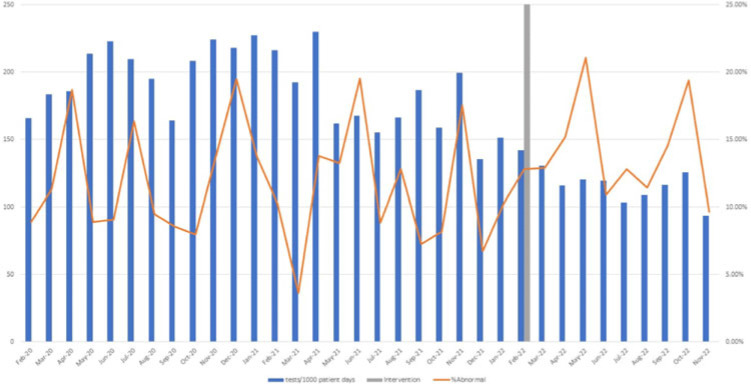

**Figure:**
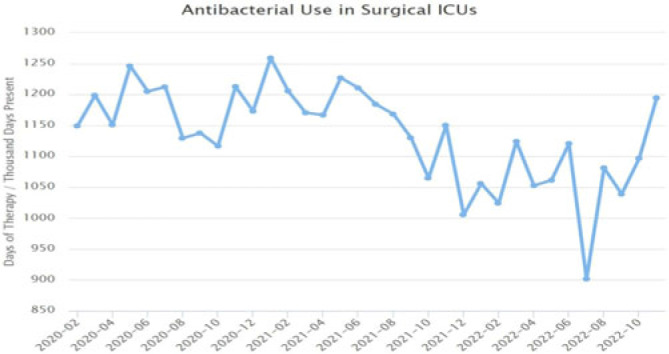

**Figure:**
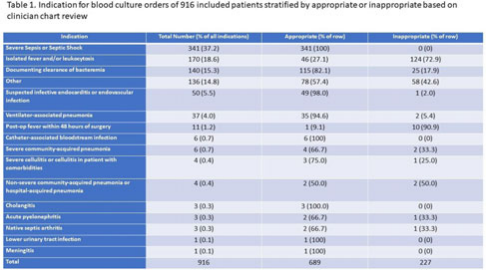

**Disclosure:** None

